# Supplementary material for: Effect of Fermentation Parameters on Natto and Its Thrombolytic Property
Source: Foods. 2021 Oct 22;10(11):2547. doi: 10.3390/foods10112547 (PMC8620952; doi:10.3390/foods10112547)
Supplement: Supplementary file 1 [file foods-10-02547-s001.zip › foods-1401994-supplementary.pdf]

Table S1 PB experiment results

Number	A (%)	B (%)	D (°C)	E (h)	G	J (%)	L (h)	NK[1] (FU/g)	FAN[2] (g/Kg)	Sensory score
1	1	3	34	30	1：1	8	1	127.23±1.39	3.50±0.02	81.3
2	1	1	34	30	2：1	6	3	135.23±2.73	4.94±0.11	83.7
3	1	1	37	24	1：1	6	3	129.50±1.45	4.55±0.09	77.7
4	0	3	37	30	1：1	8	3	130.45±2.11	7.07±0.23	79.3
5	1	3	34	24	1：1	6	1	124.89±1.59	3.50±0.06	85.7
6	0	1	37	30	1：1	6	1	136.73±3.02	9.03±0.17	80.7
7	1	1	37	30	2：1	8	1	127.95±0.78	6.51±0.16	76.3
8	1	3	37	24	2：1	8	3	118.67±0.83	3.47±0.20	69.0
9	0	3	34	30	2：1	6	3	138.45±1.27	5.95±0.07	87.3
10	0	1	34	24	1：1	8	3	142.78±3.36	6.62±0.15	72.3
11	0	1	34	24	2：1	8	1	130.06±2.48	6.16±0.17	73.7
12	0	3	37	24	2：1	6	1	122.78±1.77	6.23±0.09	75.3

[1]NK=Nattokinase

[2]FAN=free amino nitrogen content

Table S2 response surface experimental results

Number	Fermentation time (h)	Fermentation temperature (°C)	inoculation volume (%)	NK[1](FU/g)	FAN[2](g/Kg)	Sensory score
1	36	37	6	140.61±2.51	8.96±0.27	67.6
2	30	37	4	133.5±1.54	5.88±0.11	74.0
3	30	34	6	128.5±2.32	4.69±0.07	87.6
4	24	37	6	128.17±1.33	4.2±0.04	79.4
5	30	37	8	130.22±0.98	5.74±0.19	81.8
6	36	31	6	141.39±3.11	5.46±0.28	81.6
7	30	34	6	130.17±1.62	5.81±0.08	84.6
8	24	34	8	134.45±2.65	4.24±0.18	78.4
9	30	34	6	130.22±1.61	5.77±0.24	85.8
10	30	31	4	135.34±2.08	4.52±0.25	82.0
11	30	34	6	138.34±1.70	5.6±0.18	83.2
12	30	34	6	135.67±2.13	5.67±0.21	85.2
13	24	34	4	139.17±3.34	5.95±0.23	70.8
14	30	31	8	128.95±0.88	5.34±0.16	78.8
15	24	31	6	136.11±1.97	4.26±0.05	77.8
16	36	34	4	153.23±3.04	9.59±0.33	64.8
17	36	34	8	143.34±2.15	8.35±0.24	67.2

[1]NK=Nattokinase

[2]FAN=free amino nitrogen content

Table S3 analysis of experimental variance with Nattokinase Activity, amino acid nitrogen and sensory scores as response value

Source	Nattokinase Activity						amino acid nitrogen						sensory scores					
	Sum of squares	df	Meansquare	F-value	P	Significance	Sum of squares	df	Meansquare	F-value	P	Significance	Sum of squares	df	Meansquare	F-value	P	Significance
Model	607.08	9	67.45	5.52	0.0174	*	38.12	9	4.24	9.08	0.0041	**	755.76	9	83.97	18.65	0.0004	**
A- Fermentation time	206.76	1	206.76	16.92	0.0045	**	23.50	1	23.50	50.39	0.0002	**	79.38	1	79.38	17.63	0.004	**
B- Fermentation	10.79	1	10.79	0.88	0.3787		3.38	1	3.38	7.25	0.031	*	37.85	1	37.85	8.41	0.023	*
C- inoculation volume	73.69	1	73.69	6.03	0.0437	*	0.64	1	0.64	1.38	0.2783		26.64	1	26.64	5.92	0.0452	*
AB	12.82	1	12.82	1.05	0.3398		3.17	1	3.17	6.79	0.0351	*	60.84	1	60.84	13.51	0.0079	**
AC	6.68	1	6.68	0.55	0.4836		0.06	1	0.06	0.12	0.7408		6.76	1	6.76	1.5	0.2601	
BC	2.42	1	2.42	0.2	0.6698		0.23	1	0.23	0.49	0.5048		30.25	1	30.25	6.72	0.0358	*
A^2	222.39	1	222.39	18.2	0.0037	**	3.70	1	3.70	7.93	0.0259	*	323.47	1	323.47	71.84	< 0.0001	**
B^2	45.23	1	45.23	3.7	0.0958		2.21	1	2.21	4.75	0.0657		0.03	1	0.03	0.0068	0.9368	
C^2	30.69	1	30.69	2.51	0.157		1.45	1	1.45	3.11	0.121		162.64	1	162.64	36.12	0.0005	**
Residual	85.52	7	12.22				3.26	7	0.47				31.52	7	4.5			
Lack of Fit	14.77	3	4.92	0.28	0.8391		2.40	3	0.80	3.71	0.1191		21.07	3	7.02	2.69	0.1816	*
Error	70.75	4	17.69				0.86	4	0.22				10.45	4	2.61			
Sum	692.6	16					41.39	16					787.28	16				

Note: ** indicates P<0.01, * indicates P<0.05
